# AlphaFold Prediction of Protein–Protein Interactions in the *Flaviviridae* Proteomes

**DOI:** 10.3390/ijms262010159

**Published:** 2025-10-19

**Authors:** Wahyu Surya, Justin Goh, Caleb Ponniah, Jaume Torres

**Affiliations:** School of Biological Sciences, Nanyang Technological University, 60 Nanyang Drive, Singapore 637551, Singaporejgoh160@e.ntu.edu.sg (J.G.); caleb001@e.ntu.edu.sg (C.P.)

**Keywords:** *Flaviviridae* family, pestiviruses, hepaciviruses, flaviviruses, non-structural proteins, AlphaFold

## Abstract

The family *Flaviviridae* is divided into flaviviruses, hepaciviruses and pestiviruses. Its members infect a wide range of organisms, from insects to humans, and share a similar genome organization where proteins require sequential cleavage from a single translated polyprotein. Despite decades of study, the structures of some non-structural (NS) membrane proteins, or details of their protein–protein interactions (PPIs), are still unclear. Since AlphaFold (AF) can be used to predict interactions between protein domains using Predicted Alignment Error (PAE) score plots, we hypothesized that AF-predicted interactions between domains of complete viral polyproteins can represent PPIs if these interactions are retained once the different proteins are sequentially cleaved. We complemented this approach using AF predictions involving all independent separate protein sequences, instead of using a single polyprotein. We found that most of these PPIs have already been reported experimentally, which validates the use of AF in this context, but not all of these PPIs have been characterized from a structural perspective. Thus, we propose that AF provides testable hypotheses regarding residues involved in these PPIs, and that comparison of the three genera in this family may provide much needed clues to the function of these proteins during the viral life cycle.

## 1. Introduction

Viruses of the *Flaviviridae* family are responsible for significant morbidity and mortality worldwide. These small, enveloped RNA viruses are roughly distributed in the three genera *Hepacivirus*, *Flavivirus* and *Pestivirus*. Despite efforts in the last few decades, our understanding of the protein roles in these viruses is in some cases incomplete, especially for membrane proteins that do not have an enzymatic or structural role, such as non-structural (NS) proteins NS2 or NS4. In some cases, even the number of transmembrane domains (TMDs) is still debated; for example, flavivirus NS4B has five ‘predicted’ transmembrane (pTM) hydrophobic domains [1,2,3,4]. However, whereas in DENV only the last three may be membrane-spanning TMs [1], five ‘true’ TM domains have been reported for ZIKV NS4B in transfected cells [5], where the C-terminus has a cytoplasmic orientation. In addition, the structure and function of these proteins, and their involvement in protein–protein interactions (PPIs), is still uncertain. Part of the characterization problem may originate from protein behavior differences in the context of infected cells versus transfected cells, where most studies are performed. Predicting these PPIs may guide future experimental work, or provide insights for their roles in the life cycle and new avenues for the development of antivirals.

**Figure 1:**
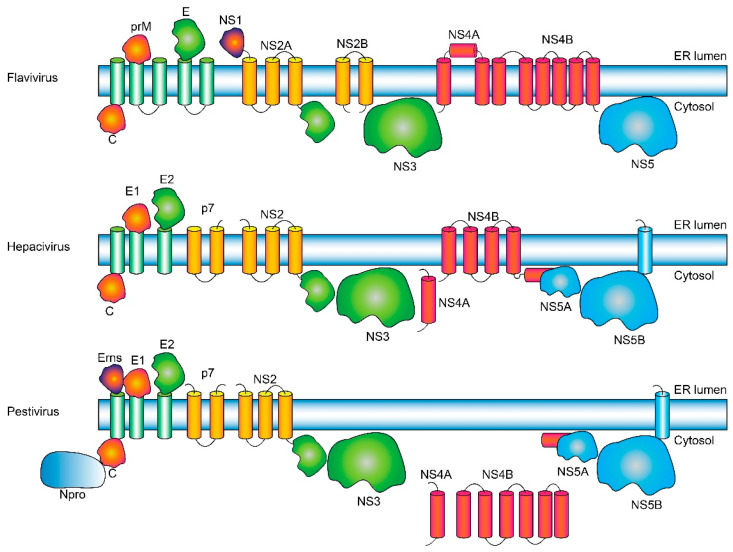
Putative topology of *Flaviviridae* polyproteins. Schematic representation of the proteins and topology (ER lumenal versus cytoplasmic) in flaviviruses, hepaciviruses and pestiviruses are indicated. We note that the precise number of TMDs is sometimes species-dependent (e.g., NS4B in flaviviruses), and in some cases it is not even firmly established. Since neither NS4A (in hepaciviruses) nor NS4A or NS4B (in pestiviruses) have any predicted TMDs according to TMHMM or Deep TMHMM, we have placed them outside the membrane. For clarity, the viral/host enzymes involved in cleavage [27] are not shown.

The genome in the *Flaviviridae* is a single-stranded positive-sense RNA molecule that is translated by the host cell machinery. The different viral proteins are obtained by a meticulously choreographed cleavage by both viral and cellular enzymes of a single translated polyprotein. The individual viral proteins present some significant differences among the three genera (Figure 1). In the polyprotein, structural proteins that will form the virion, e.g., core (capsid C in flaviviruses) and envelope (E) glycoproteins, are located N-terminally. The C protein binds RNA and triggers viral envelope formation and budding into endoplasmic reticulum (ER)-derived membranes. These membranes contain the envelope (E) glycoproteins that form a highly ordered array on the surface of the mature virion: E1 and E2 (hepaciviruses), Erns, E1 and E2 (pestiviruses), and prM and E (flaviviruses) [6,7]. Virions then transit from the ER lumen to the cell surface via the secretory pathway.

In flaviviruses, the prM protein protects the fusogenic E protein during transit [8]. In contrast, hepaciviruses and pestiviruses mature rapidly after their formation [9]; they encode for a similar protein, p7, that also has channel activity [10,11,12,13,14,15] and is found in the infected cell also as uncleaved E2-p7 polypeptide.

NS proteins have either enzymatic activity (e.g., NS3 and NS5) or roles in replication and morphogenesis [16,17], and they have genus-dependent significant differences. In general, NS proteins are crucial in immune system evasion and in the formation of the replication complex (RC) [18,19,20], a virus-induced membrane network derived from the endoplasmic reticulum (ER) [21,22,23] where viral RNA is synthesized [24,25,26]. For example, in flaviviruses, NS1 is present, but not in other members of the family. Also, NS2 is split into NS2A and NS2B (NS2 is a single protein in other genera), whereas NS5 is a single water-soluble protein, and both NS4A and NS4B have several TMDs. In contrast, in hepaciviruses only NS4B has predicted TMDs, whereas in pestiviruses no TMDs are predicted for either NS4A or NS4B proteins.

NS3 has viral protease (N-terminal) and helicase (C-terminal) domains [27,28] and requires an internal cofactor that modulates activity [27] (NS2B protein in flaviviruses and NS4A in the other two genera). In flaviviruses, NS5 is a viral RNA-dependent RNA polymerase (RdRp), whereas in the other two genera, enzymatic activity is performed by NS5B, whereas NS5A has no enzymatic activity. However, the overall functions of NS5 in flavivirus versus the pair NS5A/NS5B in the other genera are very similar [28,29].

Flaviviruses include vector-borne human disease agents such as yellow fever virus (YFV), Zika virus (ZIKV), dengue virus (DENV) and West Nile virus (WNV) [30,31,32]. They encode ten proteins: Capsid (C), pre-membrane/Membrane (prM/M), Envelope (E), NS1, NS2A, NS2B, NS3, NS4A, NS4B and NS5, where the four membrane-associated NS proteins (NS2A, NS2B, NS4A and NS4B) remain poorly characterized [20].

The main members of hepaciviruses are Hepatitis C virus (HCV) and GB-viruses. They also encode ten proteins: Core (C), E1, E2, p7, NS2, NS3, NS4A, NS4B, NS5A and NS5B. Finally, pestiviruses include pathogens that cause major damage to livestock [33] and are mainly represented by bovine viral diarrhea virus type 1 and 2 (BVDV-1 and BVDV-2), classical swine fever virus (CSFV) and Border disease virus (BDV) [34,35]. These have been recently renamed as pestivirus A–D, respectively [36]. They produce at least 12 mature proteins [37]: structural C (capsid protein, core), Erns (envelope protein, RNase secreted), E1 and E2 and eight NS proteins: N-terminal autoprotease (Npro), p7, NS2, NS3, NS4A, NS4B, NS5A and NS5B) [27,38], where NS3 to NS5B are necessary for viral RNA replication [39].

We hypothesized that interactions between domains in the expressed polyprotein may be later retained once the different proteins are cleaved, which can be assessed by comparing AlphaFold (AF) predictions with existing experimental data confirming PPIs. AF can be used to predict interactions between proteins or between domains of proteins using the Predicted Alignment Error (PAE) score [40]. Thus, herein we compared the AF predictions using complete viral polyproteins (up to 4000 amino acids) with runs using all, or a selected number of, separate protein sequences. Most of the AF-predicted interactions reported here have been already reported experimentally, but crucially lack structural detail. We propose that the latter can be provided by AF in the form of testable hypotheses. Also, comparing results for members of the three genera of this family may provide clues to the function of these proteins during the life cycle, and a basis for analysis of other similar polyproteins in other viruses.

## 2. Results

The PPIs for the proteins in members of the Flaviviridae family were obtained both using polyproteins (ranging approximately from 3000 to 4000 amino acids, or using all protein sequences as separate proteins, using AF3 (see Section 3). Results are expressed as PAE plots since they represent confidence in the orientation of domains in proteins.

### 2.1. Flavivirus Genus

#### 2.1.1. Polyproteins in Flaviviruses

##### prM and E

**Figure 2:**
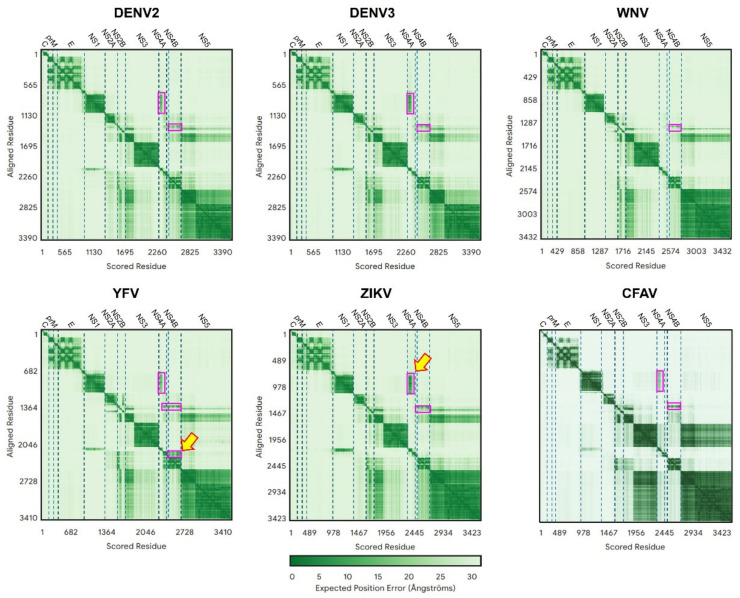
Predicted Aligned Error (PAE) for the polyproteins in six representatives of the genus flavivirus. In this and other similar figures, protein names are indicated at the top of the panel and are delimited by vertical dotted lines. Regions of low PAE values (darker green) predict PPIs. Values on both sides of the diagonal are identical. Yellow arrows and purple rectangles are shown to guide the eye to some of these PPIs.

In flaviviruses (YFV, WNV, DENV2, ZIKV and DENV3) (Figure 2), PAE values were clearly lower (darker green) when connecting prM (especially its N-terminal domain) and E protein, which is consistent with the known interaction between these two proteins [41,42].

##### NS1 and NS4A

Interactions were also predicted between NS1 and NS4A, more evident in DEN3 or ZIKV, but not in WNV.

##### NS2B and NS4

Also predicted is an interaction between NS2B, the cofactor of NS3 protease domain, and the C-terminal domain of NS4A and NS4B proteins, especially observed in FYV.

##### NS3/NS5 and NS4A

Lastly, the N-terminal domains of NS3 and NS5 (both cytosolic, Figure 1) [43,44] are predicted to interact between them, and with NS4A. 

##### NS4A and NS4B

Lower (darker) PAE values (representing likely interaction) were observed between NS4A and NS4B (especially in YFV, Figure 2).

#### 2.1.2. Separated Proteins in Flaviviruses

**Figure 3:**
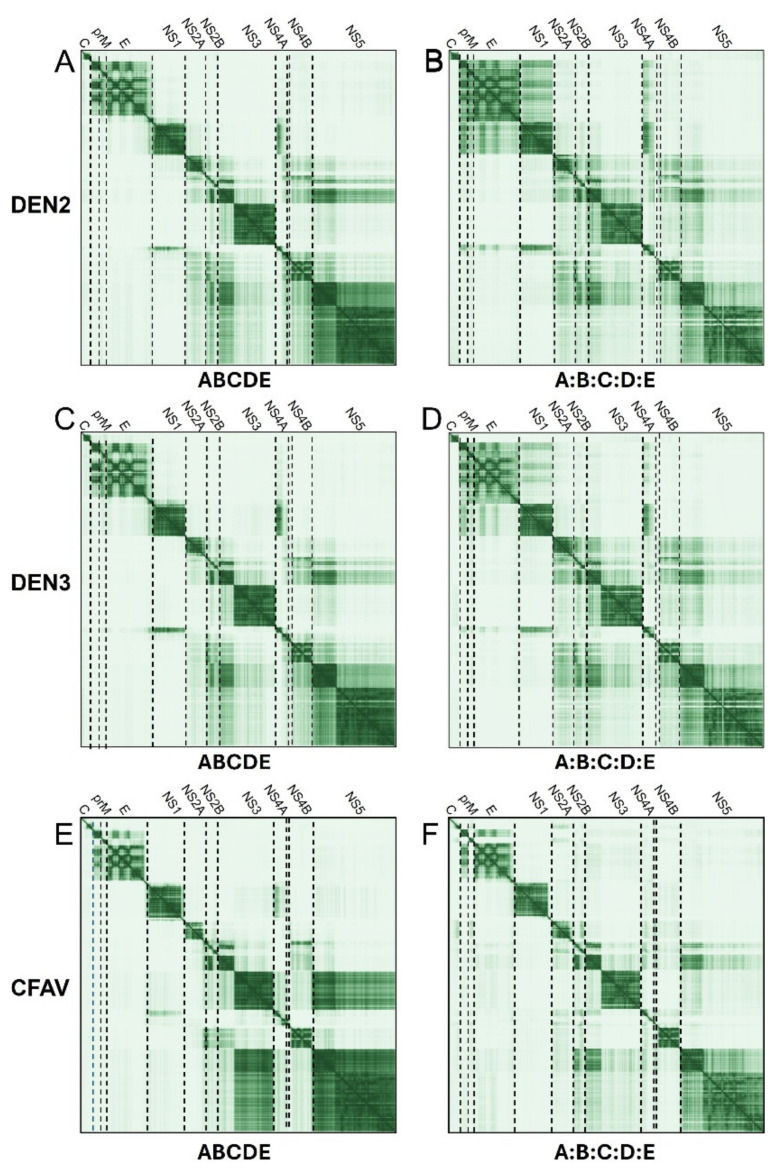
PAE plots for polyprotein versus separate proteins in flaviviruses. Polyprotein results (**left column**) are compared to separated proteins (**right column**) for DENV2 (**A**,**B**), DENV3 (**C**,**D**) and CFAV (**E**,**F**).

We then tested the AF predictions when using independent (cleaved) proteins, instead of a single polyprotein. For simplicity, the side-by-side comparisons of the PAE plots for these two forms are only shown for three flavivirus species: DENV2, DENV3 and a distant member of the flavivirus genus, Cell-Fusing Agent Virus (CFAV) (Figure 3).

In DENV2 or DENV3, the most striking difference between using polyprotein and using separate proteins is the lower PAE values (darker) between prM, E, NS1 and NS4A observed when using separate proteins (see Figure 3, comparison of panels BD and AC) These interactions were not detected in CFAV (Figure 3E,F), but a faint interaction between C and NS2A was apparent. A weaker interaction is also observed between NS5 and NS3, whereas NS2A and NS2B (especially the latter) have predicted contacts with the N-terminal domains of NS3 and NS5, NS4B and possibly NS4A. NS4B is predicted to interact with the N-terminal part of NS2B. In CFAV (Figure 3E,F), NS5 interacts with the C-terminal helicase domain of NS3, and similarly to DENV, NS2B interacts with the N-terminal domain of NS3 and NS4B, whereas NS1 interacts with the N-terminal domain of NS4A.

#### 2.1.3. Detailed Interactions

**Figure 4:**
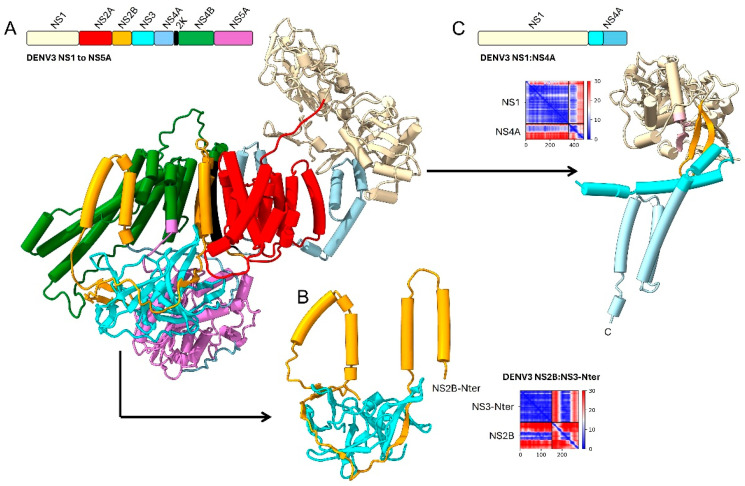
Details of predicted interactions in flaviviruses (DENV3, as an example). (**A**) AF3-predicted polyprotein structure from NS1 to NS5, where the C-terminal domains of NS3 and NS5 (residues after 250 amino acids) have been removed for clarity; (**B**) interaction between NS2B (orange) hydrophilic loop linking the two predicted TMDs, and NS3 (cyan) N-terminal domain (its first 150 residues); (**C**) interaction between NS1 (gold) and NS4A (cyan), via the N-terminal domain of NS4A (darker cyan), where N-terminal NS4A residues Asp26 and Arg39 and NS1 residues Asp35, Asp155, Glu154 and Thr162 are involved in PPIs. In (**B**,**C**), PAE plots for these interactions were obtained using AF2, where low PAE (likely interaction) is shown as blue.

Some of these AF-predicted interactions are shown in detail for DENV3 proteins NS1 to NS5A (Figure 4), mostly occurring among TMDs (Figure 4A) However, others involve hydrophilic domains: (i) between the N-terminal domains of NS3 and NS5, (ii) between a hydrophilic loop of NS2B and the N-terminal domain of NS3 (Figure 4B) and (iii) between NS1 and the N-terminal domain of NS4A (Figure 4C).

### 2.2. Hepacivirus Genus

#### 2.2.1. Polyproteins and Separate Proteins in Hepaciviruses

**Figure 5:**
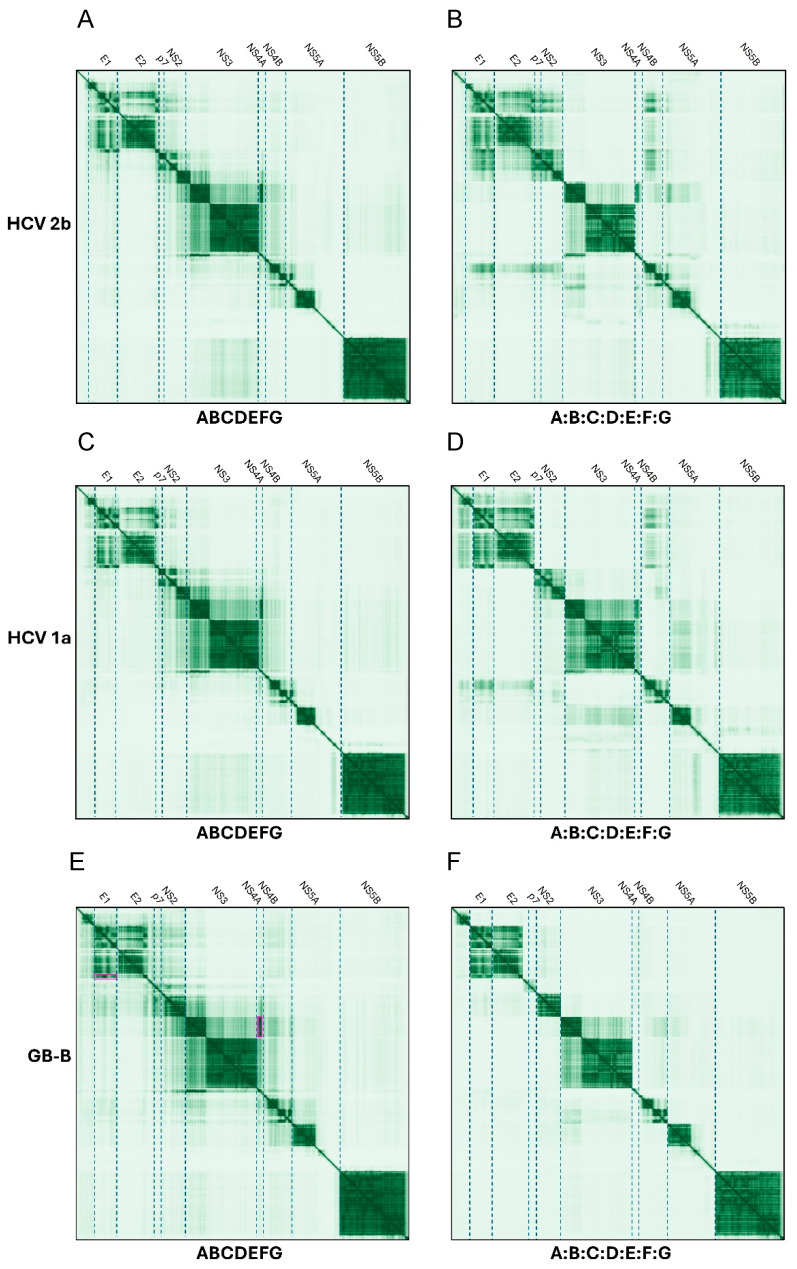
Comparison of PAE plots for polyprotein versus separate proteins in hepaciviruses. Polyprotein results (**left column**) are compared to separated proteins (**right**) for HCV 2b (**A**,**B**), HCV 1a (**C**,**D**) and GB-B (**E**,**F**).

For hepaciviruses, PAE plots were used to compare predictions using polyproteins or separate proteins (Figure 5).

##### E1 and E2, p7, NS2, NS4A and NS4B

For HCV genotype 2b (Figure 5A,B) E1 was predicted to interact with E2, and possibly p7, NS2 and NS4B. NS4A was predicted to interact with the N-terminal domain of NS3 via its C-terminal β-strand. HCV NS4B was predicted to interact with the N-terminal domain of NS5A. When proteins were considered separately, NS4B also formed interactions with E1, NS2 and p7 (Figure 4B). These interactions (e.g., between NS2 and E1) are stronger (darker PAE) when using separated proteins in DENV genotype 2b.

**Figure 6:**
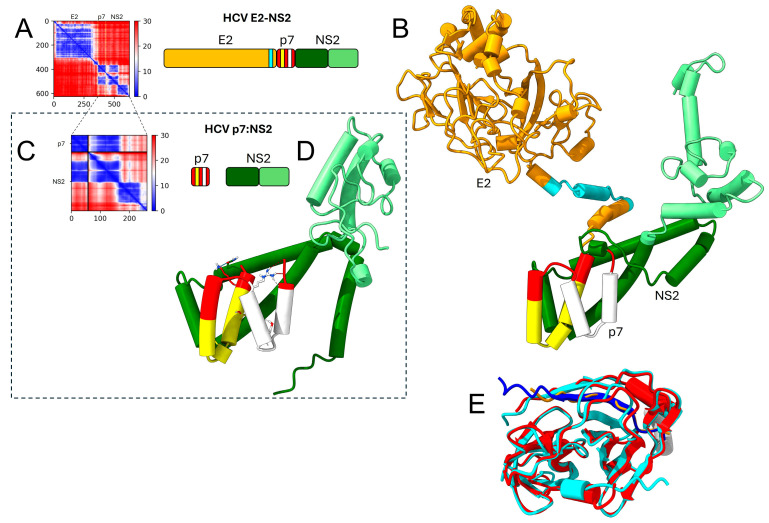
Details of some of the AF2 predicted interactions for hepacivirus HCV in Figure 5. (**A**–**D**) Predicted interactions in the segment E2 to NS2, showing PAE plot obtained in AF2 and color-coded proteins involved, with highlighted TM domains in E2 (cyan) and p7 (yellow and white) (**A**), predicted structure of the polypeptide (**B**), detail of the interaction p7/NS2 using separated proteins (**C**,**D**) and detail of interaction between the N-terminal domain of NS3 and NS4A (**E**), where NS4A intercalates within a β sheet of the enzyme core [45] in the crystal structure (PDB 1JXP) [46]. AF-predicted and experimental model have a RMSD of ~0.7 Å. It is noteworthy that instead of the predicted short helical hairpins for NS4B, p7 or NS2B, AF always predicts straight TMD helices for NS4A.

In this genus, p7 is predicted to interact with the N-terminal domain of NS2 (see details of this interaction in Figure 6), both when using a single polypeptide or as separate proteins, but there was no predicted interaction between p7 and E2, or between NS3 with p7 and NS2.

### 2.3. Pestivirus Genus

**Figure 7:**
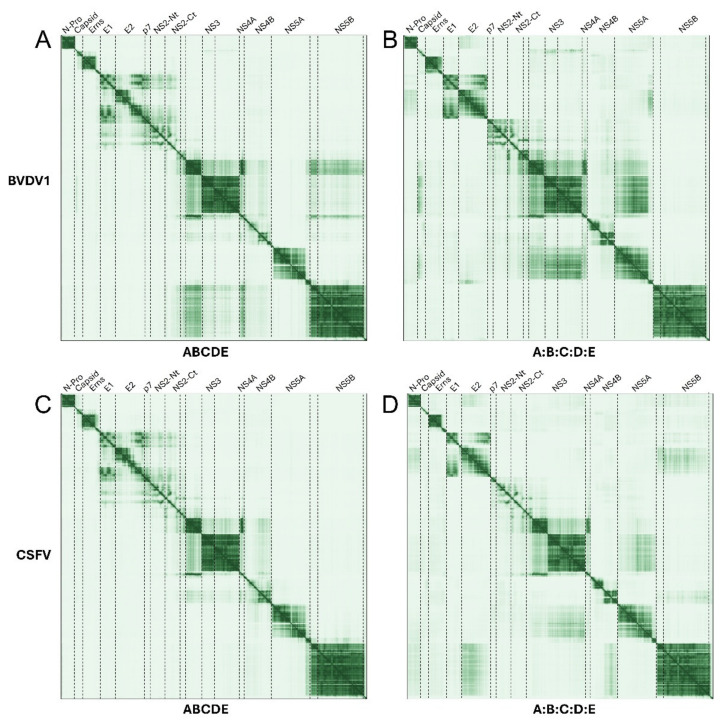
Comparison of PAE plots for polyprotein versus separate proteins in pestiviruses. Polyprotein results (**left column**) are compared to separate proteins (**right**) for BVDV1 (**A**,**B**) and CSFV (**C**,**D**).

For pestiviruses, PAE plots were used to compare predictions using polyproteins or separate proteins in BVDV1 and CSFV (Figure 7).

#### 2.3.1. Polyprotein and Separate Proteins in Pestiviruses

##### NS4A and NS3

The interaction between NS4A and the N-terminal domain of NS3 (peptidase) is predicted in all panels, and possibly the latter with NS5B (in Figure 7A, BVDV1 polyprotein).

##### E1, E2, p7 and NS2

There is also a clear predicted interaction between E1, the C-terminal domain of E2, p7 and possibly NS2, but the last two are only clear when using the polyprotein (Figure 7A,C). When using separate proteins, p7 seems to interact with NS2 (Figure 7B,D) and a complex is predicted between p7, NS2, NS3 and NS4A (Figure 7B).

##### NS5 and NS3

NS5A is predicted to interact with the C-terminal helicase domain of NS3.

##### C and NS3

In BVDV1, there is a possible interaction of C with NS3 (Figure 5B).

#### 2.3.2. TM Predictions

**Figure 8:**
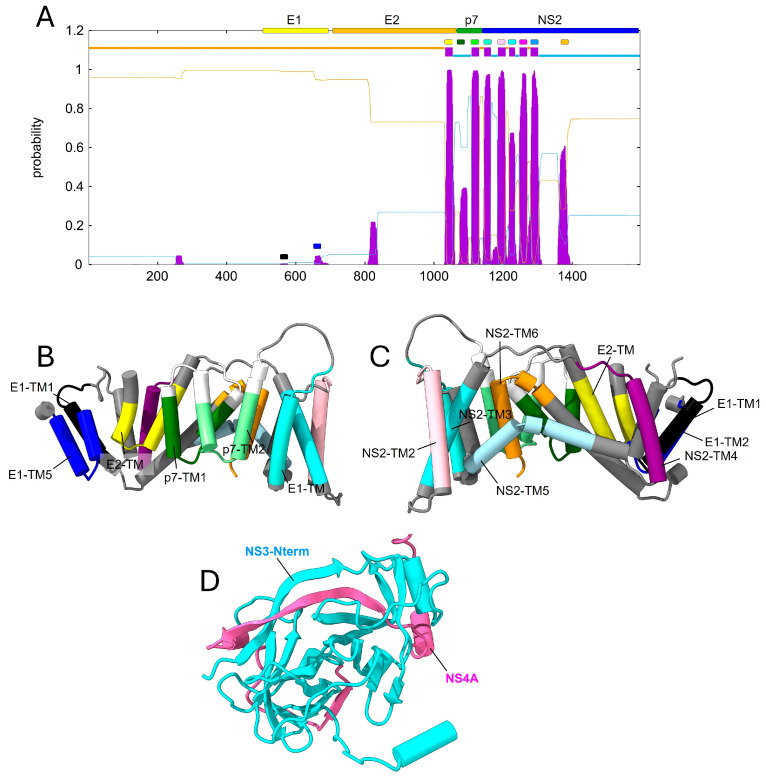
AF2 prediction for N-terminal to NS2 in pestivirus BVDV1 polyprotein. (**A**) Prediction of TMDs in TMHMM for the polypeptide N-terminus to NS2 protein. TMDs are color-coded; (**B**,**C**) interaction between the TMDs predicted in (**A**). Other residues have been omitted for clarity. E1 has two hydrophobic segments (black and blue) but are not predicted to be TMDs in TMHMM. E2 has one TMD (C-terminal, yellow) and NS2 has six predicted TMDs with another hydrophobic fragment (orange), also shown in (**B**,**C**); (**D**) detail of the interaction between N-terminal domain of NS3 (cyan) and NS4A (pink), similar to what is found in hepaciviruses.

A TMD prediction of the polyprotein in BVDV1 shows that the first TMD is located C-terminally of E2, followed by the TMDs of p7 and by the six TMDs in NS2 (Figure 8B,C). However, most predicted TMDs appeared to form a helical hairpin instead of a regular α-helix, except TM2 and TM3 of NS2. Finally, the interaction between NS4A and the N-terminal domain of NS3 is also predicted (Figure 8D).

## 3. Discussion

In this work, PPIs for the proteins in members of the *Flaviviridae* family were predicted by AF3 either using the complete polyproteins as input or using all the proteins in the genome as separated sequences for input. We note that AF3 has been previously used to successfully predict the structures of individual components of a 10 MDa pyruvate dehydrogenase complex, where it was observed that predictions of individual components were more accurate in the presence of other complementary subunits, highlighting the importance of binding partners in accurate predictions of complex structure [47].

In flaviviruses, we observed predicted interactions between prM (especially its N-terminal domain) and E, consistent with their experimentally known interaction [41,42].

NS3 and NS5 also interact, especially through their N-terminal domains, and this interaction has been reported in infected cells [43].

We also observed interactions between NS1 and NS4A, also reported experimentally [48]. Since NS1 is water-soluble and has a lumenal (exo) orientation, it is expected that this interaction involves a lumenally oriented part of NS4A. One possible candidate is the N-terminal hydrophilic domain of NS4A. Alternatively, despite NS4A having three predicted TMDs (pTMs) in most flaviviruses, a ‘U-shaped’ form has been proposed from experiments in transfected cells and purified protein in micelles [25,49], where the second pTM does not traverse the membrane and would be exposed lumenally since both N- and C-termini face the cytoplasm (Figure 1). Somewhat consistent with this proposed ‘U-shape’, in more distant insect-specific flaviviruses, like Cell-Fusing Agent Virus (CFAV) or Kamiti River virus (KRV), NS4A only has two predicted TMDs which are separated by a more hydrophilic extramembrane fragment (see Supplementary Figure S1). However, AF3 (and AF2) predict that the NS1-NS4A interaction involves the N-terminal extramembrane domain of NS4A, which should be cytoplasmic-oriented, since it is covalently linked to the C-terminal end of cytoplasmic NS3 (Figure 1). Such interaction demands that the NS4A N-terminal domain have a lumenal orientation, which would require flipping the first NS4A TMD across the membrane once NS4A is cleaved from NS3. In DENV, this interaction involved the NS4A-2K-NS4B precursor [50], suggesting the possibility that the topology of NS4A changes either after the NS2B-NS3 protease cleaves itself from NS4A, or after 2K-NS4B is cleaved from the NS4A-2K-NS4B polypeptide. A similar change in topology triggered by enzymatic cleavage has been observed for the C-terminus of NS4B, which becomes oriented luminally after being released from NS5 by viral NS2B/NS3 protease [1]. Mature NS4B is produced after NS4A, and later 2K, are cleaved from this construct [25,51]. The latter step has been reported to trigger membrane rearrangements [25,52,53,54,55], which again suggests that cleavage events are associated to large conformational changes, possibly changed topologies.

The interaction between NS2B and NS4 is expected from the knowledge that NS2B-NS3 cleaves NS4A from the intermediate NS4A-2K-NS4B at the cytosolic side of the ER membrane, and in DENV, the interaction between NS2B-NS3 and NS4A-2K-NS4B has been linked to de novo formation of vesicle packets (VPs), where RNA replicates [56]. Further, in infected cells, the interaction between NS4A-2K-NS4B with NS1 and NS2B is required for viral replication [48,50,57,58,59], and NS4B colocalizes with, or recruits, NS1, NS2B, NS3 and NS4A, together with viral RNA [54,55,60,61,62,63,64,65,66].

The association of NS3 with NS4A and NS4B proteins is consistent with their involvement in dissociating single-stranded RNA from the NS3 helicase C-terminal domain [53,67], but interaction with this domain is not predicted here (Figure 2 and Figure 3). In DENV2, this involves NS4B TM2 (residues 51–83) [68] and the ‘cytosolic loop’ (residues 130 to 167) [53,67,69].

Finally, the predicted interaction between NS4A and NS4B, especially clear for YFV (Figure 2), has been reported experimentally in DENV, with a reported 1:1 stoichiometry and a very high affinity K_d_ of 50 nM [70,71,72]. This interaction was proposed to involve the N-terminal tail and first TM of NS4A (residues 40–76) and TM3/cytoplasmic loop of NS4B (residues 84–146) [70]. However, we note that direct interaction between flavivirus NS4A and NS4B has not been independently confirmed in vitro [73].

When using separated proteins, we observed a higher probability of interaction (lower PAE) in DENV2 or DENV3, but not in CFAV, between prM, E, NS1 and NS4A. This suggests that in this case these interactions may be constrained by the covalent linking in the polyprotein, and that they are only possible once the proteins are cleaved. CFAV showed a faint possible interaction between C and NS2A, not shown in DENV sequences. Also predicted is an interaction network involving NS5, NS3, NS2A, NS2B, NS4B and NS4A. It is known that NS3-mediated cleavage of C is facilitated by NS3 flavivirus protease cofactor NS2B [74]. NS2A binds RNA and colocalizes with replication complexes [75,76]; thus, it may shuttle genomic substrates out of membrane-bound replication complexes to the sites of packaging. The NS3 proteolytic role in assembly of flaviviruses may be similar to that of uncleaved NS2-3 in pestiviruses (see below) [74]. Flavivirus morphogenesis requires NS2A and NS3 [77], and NS3 and its cofactor NS2B for capsid protein processing [74].

In hepaciviruses, interactions were predicted for E1 and E2, p7, NS2, NS4A and NS4B. The interaction of NS4A, which has no predicted TMDs in this genus, with NS3, is consistent with its cofactor role of NS2-NS3 activity [46,78,79]. HCV NS4B was predicted to interact with NS5A. NS4B forms homo-oligomers that are important for inducing the membrane structure during viral replication [80], and when considered separately formed interactions with E1, NS2 and p7. In this genus, p7 was predicted to interact with the NS2, but not with E2 or NS3.

In hepaciviruses (and also in pestiviruses), NS2 is a cysteine autoprotease that self-cleaves from NS3 [81] but uncleaved precursor NS2-3 is required for infectious pestivirus production, independent from enzymatic activity [78,82,83,84]. Therefore, PPIs between NS2 and NS3 are expected. The interaction between p7, E2, NS2 and NS3 has been reported in the literature [85,86,87,88,89]; p7 regulates the subcellular localization of NS2 and core protein [90], and p7/NS2 dictates the relocalization of core from LDs to ER in infected cells. In both pestiviruses and hepaciviruses, p7 has both N and C termini oriented lumenally [91] and is involved in release of infectious virions and virulence [11,82,92,93,94,95]. p7 colocalizes with E2, NS5A, C, NS2 and NS3 [96,97]. Cleavage at E2p7 is incomplete, leading to the presence of both E2 and p7 mature forms, as well as some E2p7 [98,99,100,101]. In both genera, p7 participates in virion assembly by interacting with NS2 and E2 [89,97,102,103,104,105]. In pestivirus CSFV, p7 and NS2 interact mainly through the first TMDs of each protein [102]. In HCV, the interactions of NS2 with E1, p7 and NS3 synergistically modulate virus assembly [83]. The p7 and NS2 proteins are key determinants governing the subcellular localization of the HCV C from lipid droplets (LDs) to the ER, and are required for the initiation of the early steps of virus assembly [90]. Also, CSFV NS2 has been recently reported to modulate the NS3/4A-kink interaction [106].

In pestiviruses, we observed possible interactions between NS4A and NS3, confirmed experimentally [79], and also between E1, the C-terminal domain of E2, p7 and possibly, NS2, and a complex is predicted between p7, NS2, NS3 and NS4A, as well as NS5A with NS3 and C with NS3. Interestingly, most predicted TMDs appeared to form a helical hairpin instead of a regular α-helix, except for TM2 and TM3 of NS2.

Overall, we have used low PAE values to estimate a likelihood for local and global coordinate positions. This approach is validated here simply by the fact that most, if not all, PPIs described herein have been reported experimentally. However, the detailed models obtained may not be entirely correct or unique. Instead, they can be used to generate testable hypotheses. The general cases of pitfalls during evaluation of AF2 model quality using PAE values used herein have been recently discussed [107]. Another difficulty is that many of the proteins in the polyprotein contain hydrophobic domains, which are much less represented in the AF training sets.

## 4. Materials and Methods

### 4.1. Sequences Used in AF Structure Prediction

The sequences used for flaviviruses corresponded to WNV (3434 aa), accession number EMBL BBD13921 and Uniprot/Uniparc (universal identifier UPI000DBB6F5B), YFV (3411 aa), accession number EMBL XER92105 and Uniprot/Uniparc (universal identifier UPI003A5D6927), ZIKV (3423 aa), accession number EMBL QOF88708, Uniprot/Uniparc (universal identifier UPI0018A44B63), DEN2 (3391 aa), accession number EMBL identifier WLD15673, Uniprot/Uniparc (universal identifier UPI00291CEA53) and DEN3 (3390 aa), accession number EMBL identifier WGL08553, Uniprot/Uniparc (universal identifier UPI0027A65906). The sequences used for hepaciviruses were HCV genotype 2b (3033 aa), accession number EMBL BAJ07247, Uniprot/Uniparc (universal identifier UPI0001D25ED9) and HCV genotype 1a (3011 aa), accession number EMBL ACH61709, Uniprot/Uniparc (universal identifier UPI00017FB688). For pestiviruses, we used H GB-B (2864 aa), we used accession number EMBL AAF01368. In Uniprot/Uniparc (universal identifier UPI0000035CCD), BBDV1 (3898 aa), we used EMBL accession number XWV28335.1, Uniprot/Uniparc (universal identifier UPI00017FB688).

### 4.2. AF3 Server

Structure prediction of viral polyproteins (as a single polypeptide), and of processed individual proteins in the polypeptide, was obtained in AF3 server and a ColabFold (AF2) notebook, respectively. No significant differences were observed between the two methods when comparing smaller subsets of the polyprotein. Results shown herein were obtained with AF3, except when indicated otherwise. AF3 prediction was performed using the AlphaFold Server (https://alphafoldserver.com) [40] (accessed on 14 May 2025). After the process was finished, we selected the top-ranked model. PAE Viewer web server was used to generate and evaluate PAE plots [108].

### 4.3. ColabFold Notebook

ColabFold (ColabFold v1.5.5: AlphaFold2 [109,110]) (Colab Pro+ subscription) was used in predictions involving a subgroup of the proteins in the polyprotein, typically with a total number of amino acids lower than 2500. We used the parameters, unless otherwise specified: no templates, 6 recycles (forced to complete 6 with ‘recycle_early_stop_tolerance = 0′) and one seed, which resulted in a total of five models for each run, ranked sorted by pTM score. The last model after each 6 recycles was used [111]. We used the default MMseqs2 multiple sequence alignment (MSA), which produced MSAs with >100 sequences, sufficient for a reliable prediction (<30) [110]. plDDT scores 100–90 indicate high accuracy comparable to high-resolution structures; regions with scores 70–90 are modelled well with good backbone prediction; regions of scores 50–70 are low confidence and regions <50 cannot be interpreted or may be disordered [110,112,113]. For each prediction, the best models were sorted by the predicted template modelling (pTM) score (0–1). pTM is based on a superposition of the predicted structure and a hypothetical true structure, where pTM > 0.5 means high similarity. The predicted aligned error (PAE) (measured in Å and capped at 31.75 Å) indicates the expected positional error at residue x if the predicted and actual structures are aligned on residue y. Thus, low PAE values (in ColabFold PAEs are colored generally in blue) between two domains or subunits represent well-defined relative positions and orientations of these two bodies. The best model in each run was energy minimized by OpenMM/Amber (relax_amber.ipynb), using default values 2000 max_iterations, tolerance 2.39 and stiffness 10 [109]. Molecular graphics and analyses performed with UCSF ChimeraX [114,115], developed by the Resource for Biocomputing, Visualization, and Informatics at the University of California, San Francisco, with support from National Institutes of Health R01-GM129325 and the Office of Cyber Infrastructure and Computational Biology, National Institute of Allergy and Infectious Diseases.

## 5. Conclusions

In conclusion, we have shown that AF can successfully predict interactions between proteins in the Flaviviridae family, most of which have been already confirmed experimentally, whether using a complete polyprotein as input, or using all protein sequences separately. Differences between these two outputs are likely due to (i) more freedom of movement when proteins are separated, if geometry rearrangements are needed with respect to the full-length polyprotein after the proteins are sequentially cleaved, and (ii) a reduction in entropy, and the higher likelihood of native interaction prediction, if the final interaction is still maintained after protein cleavage steps. Within this favorable context, some of the detailed interactions proposed still lack experimental confirmation, or confirmation is under debate. These predicted interactions can guide future experiments to provide additional support. Overall, three interaction networks are predicted by AF3 to exist in flaviviruses, involving (i) prM, E, NS1 and NS4A, (ii) NS2A, NS2B, NS3, NS5 and NS4A, NS4B and (iii) C, NS2A and NS2B. In hepaciviruses, the prediction points to an interaction network involving all proteins except NS5B, whereas in pestiviruses, interactions involve all proteins except water-soluble NS4B and NS5B.

It is also interesting to note that many of the TMDs predicted do not fold as regular α-helices, but as short helical hairpins. Given the abundance of these predictions within this family, we propose that they may be real features and not AF artifacts. Indeed, the prediction of hairpins in transmembrane α-helical domains is not an inherent bias of AF. For example, we have shown previously that this is not the case for NS4A in flaviviruses [73], for the envelope protein in coronaviruses [111], or bacterial aquaporins [116], where predicted α-helical transmembrane domains show perfectly straight α-helices. Instead, we propose that these structures are related to the membrane modulation properties of some or most of these proteins.

Another source of variation in this work is the difference observed between species belonging to the same genus. Prediction differences between species may represent real variations or may be caused by the slightly different Multiple Sequence Alignments (MSAs) used in each case.

## Data Availability

The datasets generated during and/or analyzed during the current study are available from the corresponding author on reasonable request.

## Supplementary Materials

The following supporting information can be downloaded at: https://www.mdpi.com/article/10.3390/ijms262010159/s1. Reference [117] is cited in the Supplementary Materials.
