# Supplementary material for: AlphaFold Prediction of Protein–Protein Interactions in the Flaviviridae Proteomes
Source: Int J Mol Sci. 2025 Oct 19;26(20):10159. doi: 10.3390/ijms262010159 (PMC12564508; doi:10.3390/ijms262010159)
Supplement: Supplementary file 1 [file ijms-26-10159-s001.zip › ijms-3898984-supplementary.pdf]

Supplementary File.

Protein-Protein Interactions in the Flaviviridae Family Predicted by AlphaFold

Wahyu Surya, Justin Goh, Caleb Ponniah and Jaume Torres

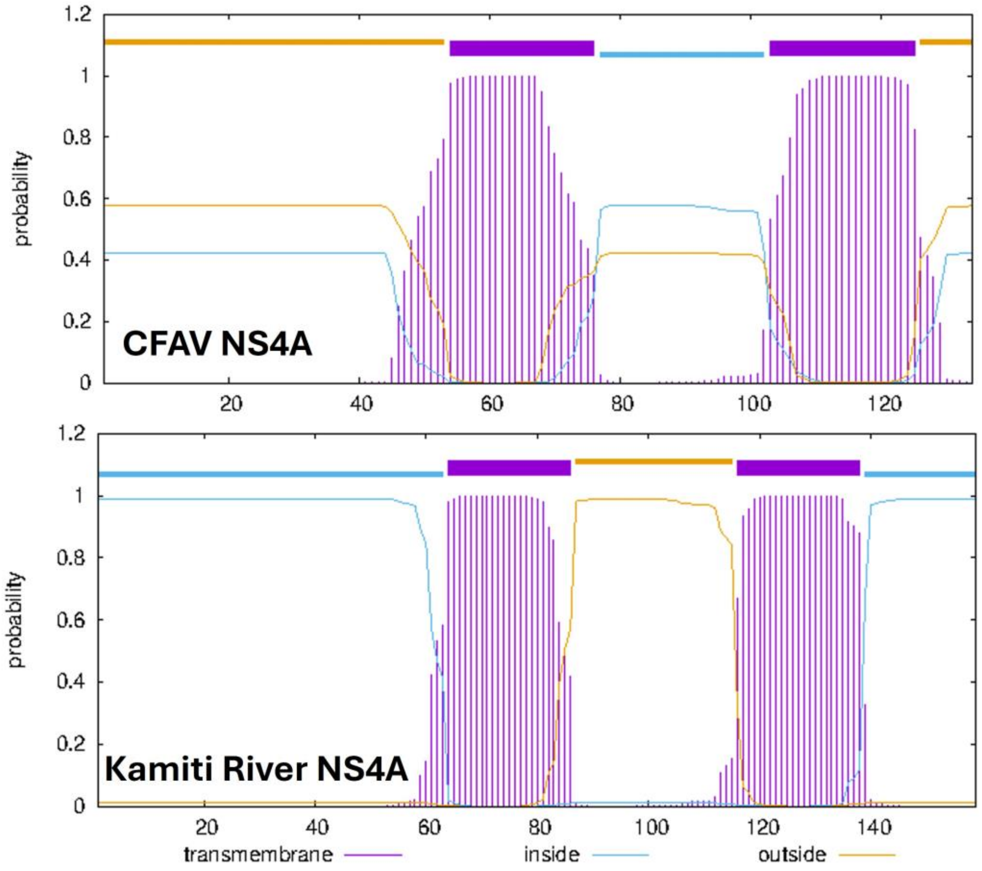

Supplementary Figure S1. TMD prediction by TMHMM [1] for NS4A protein in flavivirus CFAV and Kamiti river virus (KRV), as indicated. Although three hydrophobic stretches are predicted for NS4A in other flaviviruses, here only two are predicted with opposite topology (N- and C-terminus outside (lumenal) in CFAV and inside (cytoplasmic) in KRV. The second predicted TM in other flaviviruses is here hydrophilic.

1. Sonnhammer, E.L.; von Heijne, G.; Krogh, A. A hidden Markov model for predicting transmembrane helices in protein sequences. *Proceedings. International Conference on Intelligent Systems for Molecular Biology* **1998**, *6*, 175-182.
